# Accuracy, readability, and health information quality of AI chatbot responses for patient education on intermittent fasting in chronic kidney disease: a prospective structured evaluation

**DOI:** 10.3389/fneph.2026.1857793

**Published:** 2026-05-29

**Authors:** Joel Shah, William Wells-Gatnik, Biff Palmer, Prakrati Acharya, Wisit Cheungpasitporn

**Affiliations:** 1 Department of Internal Medicine, Texas Tech University Health Sciences Center, El Paso, TX, United States; 2 Department of Education and Internal Medicine, Texas Tech University Health Sciences Center, El Paso, TX, United States; 3 Department of Nephrology, Carl T Hayden VA Medical Center, University of Arizona, Phoenix, AZ, United States; 4 Department of Nephrology and Hypertension, Mayo Clinic, Rochester, MN, United States

**Keywords:** artificial intelligence (AI), chatbot, ChatGPT, CKD - chronic kidney disease, Claude, intermittent fasting (IF), large language model (LLM), patient education

## Abstract

**Background:**

Intermittent fasting (IF) represents a dietary strategy with known metabolic benefits, however its safety and applicability in patients with chronic kidney disease (CKD) remains poorly understood. AI chatbots’ reliability for clinical and dietary guidance in clinically complex areas remains underexplored. No study has examined AI performance on IF in patients with CKD. This study evaluated the accuracy, consistency, readability, and quality of responses generated by ChatGPT-5 and Claude 4.5 Sonnet regarding IF in CKD.

**Methods:**

Twenty-five patient-centered questions spanning seven clinical domains were developed by two board-certified nephrologists and submitted independently to ChatGPT-5 and Claude 4.5 Sonnet twice per model. Prompts were submitted under standard and unmodified user conditions without customized system instructions to represent a zero-shot evaluation that is illustrative of typical patient use. Responses were evaluated for accuracy, consistency (comparatively between responses), and appropriateness by the evaluators. Response readability and quality were assessed using the Flesch-Kincaid Grade Level (FKGL) formula and the DISCERN instrument respectively.

**Results:**

Both ChatGPT-5 and Claude 4.5 Sonnet demonstrated identical accuracy scores during the first question prompts with notable improvement upon re-prompting after 24-hours. Both models demonstrated an identical internal consistency at 90%. ChatGPT-5 produced responses at the middle school level, better aligned with the US adult health literacy benchmark, while Claude 4.5 Sonnet generated responses at the high school level. DISCERN scoring was comparable between chatbots indicative of good response quality. Both chatbots had similar performance regarding individual subdomains.

**Conclusions:**

Both chatbots illustrated clinically accurate and cautious responses to questions regarding IF in CKD. ChatGPT-5 responses were better suited to the average health literacy of the general United States adult population. Despite our findings, we do not recommend either chatbot be used as the primary source for clinical counseling regarding IF in CKD. It is important for clinicians to help to guide patients toward evidence-based clinical information.

## 1 Introduction

Approximately 697.5 million individuals worldwide are believed to have chronic kidney disease (CKD), which remains among the leading causes of global morbidity, mortality, and healthcare costs (1). CKD carries a clinical complexity that ties into other common comorbid conditions including but not limited to type 2 diabetes, hypertension, and cardiovascular disease, which each demand careful dietary management and individualization. Patients with CKD are recommended to restrict their dietary intake of potassium, phosphorus, sodium, and in the later stages of the disease, protein intake (2–5). This strict regimen is sensitive to dietary modifications which oftentimes are beneficial to CKD and other comorbidities, one such example being plant-based, low-protein diets (6).

Intermittent fasting (IF) is a dietary regimen that has gained considerable attention due to its known metabolic benefits including fat loss, systemic inflammatory modulation, and increases in insulin sensitivity (1, 7). These effects hold theoretical appeal for many patients with CKD, who carry concurrent metabolic diagnoses. However, the application of IF in this population remains underexplored and carries important caveats. Published guidance is limited, and identifiable risks such as accelerated protein catabolism, electrolyte instability, hypoglycemia in patients on glucose-lowering agents, and fluid dysregulation carry heightened significance in the setting of reduced renal reserve (2, 3, 8). Furthermore, the primary goal of IF, weight loss, may not be desirable among all individuals with CKD, particularly those with advanced disease or that are dialysis-dependent (9). In contrast to healthy individuals without systemic disease, patients with CKD have demonstrated limited physiologic buffering capacity, including increases in the accumulation of nitrogenous waste products and systemic acidemia from fasting induced catabolism (9). Specific electrolyte shifts, most notably hyperkalemia and hyponatremia, lack adequate compensation in the setting of impaired renal tubular regulatory mechanisms. Given the known adverse effects of fasting in CKD, IF is more than just a theoretical risk for this patient population and may be acutely harmful in the later stages of the disease. For this reason, AI guidance in this area of limited literature may be beneficial.

AI-powered chatbots have become a prominent source of health information for patients managing chronic illness (10, 11). Platforms such as ChatGPT and Claude are widely accessible and have demonstrated utility across numerous general medicine domains, including medication education and chronic disease management (11, 12). Whether they perform reliably in evidence-limited, subspecialty-specific contexts such as IF and CKD has not been established.

Prior work has evaluated AI chatbot accuracy for general CKD lifestyle and nutrition questions. Acharya et al. assessed ChatGPT 3.5, ChatGPT-4, and Bard AI against KDIGO and KDOQI guidelines, finding generally acceptable accuracy with nephrologist review (13). That study did not examine IF specifically, utilized older chatbot models, did not apply the DISCERN quality instrument, and did not employ a repeated-submission design to probe consistency or machine learning. Thus, the need for a study which addresses such gaps within the context of IF and CKD is necessary.

We conducted a structured prospective evaluation of ChatGPT-5 and Claude 4.5 Sonnet, assessing the accuracy, consistency, readability, and health information quality of their responses to 25 patient-centered questions on IF in CKD. We hypothesized that both models would demonstrate clinically adequate accuracy overall but would differ in the readability and the way in which questions with limited literature would be answered.

## 2 Materials and methods

### 2.1 Study design

This was a prospective, structured evaluation of two commercially available AI chatbots: ChatGPT-5 (OpenAI, San Francisco, CA, USA) and Claude 4.5 Sonnet (Anthropic, San Francisco, CA, USA), assessing the quality of chatbot-generated health information on IF in the context of CKD. No patient data were collected or analyzed. All prompts were generated by the investigators.

### 2.2 Question development

Twenty-five patient-centered questions were developed by two board-certified nephrologists. Questions were written in lay language calibrated to an 8th-to-10th-grade reading level to simulate the health literacy of adults with CKD seeking dietary information. Questions were organized into seven clinical domains: (1) safety and general eligibility, (2) metabolic and physiological effects, (3) CKD-specific complications, (4) comorbidity management (diabetes, hypertension, and cardiovascular disease), (5) monitoring and clinical oversight, (6) psychosocial impact and quality of life, and (7) special populations, including dialysis patients and sex-based differences in IF response (**Table 1**). Importantly, questions were developed by clinicians and adjusted to mimic that of a patient’s inquiry rather than sourced directly from a patient with CKD. The complete question set is provided in the **Supplementary Appendix**.

**Table 1:** Distribution of questions by clinical domain.

Question domain	n	% of total
Safety and General Eligibility	5	20%
Metabolic and Physiological Effects	5	20%
CKD-Specific Complications	5	20%
Comorbidity Management (Diabetes, Hypertension, CVD)	3	12%
Monitoring and Clinical Oversight	3	12%
Psychosocial Impact and Quality of Life	2	8%
Special Populations (Dialysis, Sex Differences)	2	8%
Total	25	100%

Questions developed by a nephrologist utilizing patient-appropriate lay language. Questions addressing multiple domains were classified according to primary clinical intent. CKD, chronic kidney disease; CVD, cardiovascular disease.

### 2.3 Chatbot interaction protocol

Each question was submitted independently to ChatGPT-5 and Claude 4.5 Sonnet through their respective standard user interfaces. To evaluate reproducibility, each question was submitted a second time to the same model at a minimum interval of 24 hours. No additional system prompts, custom instructions, or model modifications were applied, so that the study reflects a zero-shot evaluation under standard patient-use conditions.

### 2.4 Outcome measures

#### 2.4.1 Accuracy

Response accuracy was scored on a 0–2 ordinal scale by two board-certified nephrologists. A score of 2 indicated full concordance with PubMed-indexed literature or established nephrology guidelines (KDOQI, KDIGO). A score of 1 was given to partially accurate responses, indicating applicability to general literature regarding fasting but lacking CKD specificity or to responses correctly acknowledging limited or absent evidence on the topic. A score of 0 was assigned to all responses with inaccuracies, misleading responses, responses that did not answer the question, or to responses failing to identify important safety considerations. Responses reporting a lack of CKD-specific evidence regarding IF were credited for accuracy to promote the importance of limitations in the literature when educating patients. To minimize subjectivity due to the dual evaluator design of the study, domain questions were paired with referenced answers or guidelines derived from preexisting literature including PubMed, KDIGO, and KDOQI guidelines. All referenced literature was PubMed indexed. In order to promote internal consistency in accuracy scoring, responses were reviewed a second time after initial scoring. A composite average score was then obtained from the two individual nephrologist’s scores.

#### 2.4.2 Consistency

We defined consistency as the similarity in quality and content between repeated AI chatbot generated responses for a given question. A response was labeled consistent if it conveyed similar clinical information, context, recommendations, and safety considerations regardless of minor differences in grammar.

#### 2.4.3 Appropriateness and clinical relevance

Appropriateness was assessed by measuring responses based on four criteria: (1) directly addressed the clinical question, (2) included relevant safety warnings where warranted, (3) recommended individualized clinical consultation, and (4) avoided prescribing specific IF protocols without individualized assessment.

#### 2.4.4 Readability

The Flesch-Kincaid Grade Level (FKGL) formula was utilized for readability metrics. The FKGL formula generates an estimated US grade-level reading score based on sentence length and syllable count of responses. Each response score was compared to the US adult health literacy benchmark of 8^th^ grade (14).

#### 2.4.5 Health information quality

Health information quality assessment utilized the DISCERN instrument. DISCERN is a 16-item questionnaire that assesses health information for consumers (15). Responses were scored on a scale of 1 (low quality) to 5 (high quality) with a cumulative composite score generation. Key domains include clarity of purpose, acknowledgment of evidence limitations, balance, and description of treatment risks and benefits.

### 2.5 Data analysis

Descriptive statistics were used to summarize accuracy scores, FKGL values, DISCERN scores, and consistency rates by model and clinical domain. Domain-level accuracy was tabulated to identify areas of relative strength and weakness. Comparative analysis was conducted descriptively, given the rubric-based nature of the scoring methodology. Inter-rater reliability assessment was not performed; this limitation is addressed in the discussion section.

## 3 Results

### 3.1 Overall accuracy and consistency

Composite ChatGPT-5 and Claude 4.5 Sonnet demonstrated identical accuracy in their initial responses with an accuracy score of 42/50 (mean 1.68/2.0). The second set of responses (24 hours or more after generation of the first responses) showed improvements in accuracy by both chatbots with ChatGPT-5 scoring 46/50 (mean 1.84/2.0) and Claude 4.5 Sonnet scoring 44/50 (mean 1.76/2.0). Response consistency across repeated submissions was 90% for both ChatGPT-5 and Claude 4.5 Sonnet. Key performance metrics are summarized in **Table 2**.

**Table 2:** Comparative performance metrics: ChatGPT-5 vs. Claude 4.5 Sonnet.

Metric	ChatGPT-5	Claude 4.5 Sonnet
Accuracy Score- Session 1 (out of 50)	42	42
Accuracy Score- Session 2 (out of 50)	46	44
Consistency (%)	90%	90%
Physician Consultation Recommendation (%)	92%	88%
Evidence Gap Acknowledgment (%)	84%	88%
Mean FKGL	8.5 (Middle School)	10.2 (High School)
DISCERN Composite Score (/5)	4.2	4.1
Response Length	Short/concise	Long/comprehensive
Typical Format	Bullet-point	Prose/discursive

Performance across 25 patient-centered questions submitted twice per model (≥24-hour interval). Composite scores were averaged between both individual nephrologist’s reports. Accuracy scored 0–2 per response (maximum 50 per session). Consistency = content similarity between repeated responses. FKGL, Flesch-Kincaid Grade Level; DISCERN, validated consumer health information quality instrument.

### 3.2 Domain-level accuracy

Domain-level accuracy varied by topic for both models (**Table 3**). ChatGPT-5 and Claude 4.5 Sonnet had perfect accuracy scores (2/2) regarding specialty topic questions addressing hypoglycemia, electrolyte disturbances, blood pressure risks, cardiovascular effects, and safety or monitoring recommendations between both question sets. Regarding topics with limited disease specific evidence such as proteinuria, protein catabolism, CKD staging, dialysis related information, and medication interactions, performance was moderate.

**Table 3:** Domain-level accuracy scores by clinical topic (score range: 0–2).

Clinical domain	GPT-5 S1	GPT-5 S2	Claude S1	Claude S2
Hypoglycemia Risk	2	2	2	2
Electrolyte Disturbances (K^+^, Na^+^)	2	2	2	2
CKD Disease Staging	1	2	1	2
Protein Intake/Proteinuria	1	2	2	2
Acid-Base Balance/Metabolic Acidosis	2	2	1	2
CKD–Diabetes Comorbidity	2	2	2	2
Blood Pressure/Cardiovascular Effects	2	2	2	2
Dialysis-Specific Guidance	2	2	1	1
Medication Pharmacokinetics	1	2	1	1
Anemia	1	1	1	1
Gut Microbiome Effects	1	1	1	1
Sex Differences in IF Response	0	1	1	1
Long-Term CKD Outcomes	1	1	1	1
Circadian/Time-Restricted Feeding	1	2	1	2
Monitoring Recommendations	2	2	2	2

S1, Session 1 (initial submission); S2, Session 2 (repeat submission ≥24 hours later). Score of 2, full concordance with published literature or guidelines; 1, partial accuracy or appropriate evidence-gap acknowledgment; 0, factual error or clinically significant omission. IF, intermittent fasting; CKD, chronic kidney disease; K^+^, potassium; Na^+^, sodium.

The weakest performance for both models was observed on the question regarding sex-based differences in IF response in CKD: ChatGPT-5 scored an average of 0 in the first session and 1 in the second, while Claude 4.5 Sonnet scored 1 in both sessions. This pattern reflects the near-total absence of published literature on this topic rather than a model-specific failure. Both models also scored an average of 1 across both sessions for questions on anemia management and gut microbiome effects in CKD, consistent with the limited available evidence in these areas.

### 3.3 Consistency and interval-dependent response changes

Content-level consistency was 90% for both models on average. Differences between sessions were most common in response length and structure rather than clinical substance. Second-session responses from both models tended to include more detailed clinical caveats and more explicit acknowledgment of the limited IF-specific literature in CKD. This pattern was observed across multiple domains, including metabolic acidosis, dialysis eligibility, and medication interactions. Whether this reflects ordinary variability in language model output or some interval-dependent effect cannot be determined from this study design.

### 3.4 Readability

ChatGPT-5 produced responses at a mean FKGL of 8.5, corresponding to an 8^th^ to 9^th^ grade reading level, closely aligned with the estimated US adult health literacy benchmark. Claude 4.5 Sonnet generated responses at a mean FKGL of 10.2, corresponding to a high school sophomore level. This difference was attributable primarily to Claude 4.5 Sonnet’s tendency toward longer sentences, multi-clause constructions, and extended mechanistic elaboration. For patients with lower educational attainment, elderly patients, or those for whom English is a second language, demographics overrepresented in CKD populations, this reading level difference may have practical implications for comprehension.

### 3.5 Health information quality (DISCERN)

DISCERN composite scores demonstrated good to high quality responses by ChatGPT-5 (4.2/5) and Claude 4.5 Sonnet (4.1/5) when cross-referenced to established literature (**Table 4**). Both chatbots demonstrated relevance, balance, and clarity in their responses. The lowest sub-domain scores for both models were in ‘clarity of information source’ and ‘timeliness of information' reflecting the absence of explicit literature citations and the inability of either platform to indicate the recency of incorporated knowledge.

**Table 4:** DISCERN sub-domain scores: ChatGPT-5 vs. Claude 4.5 Sonnet.

DISCERN sub-domain	ChatGPT-5	Claude 4.5 Sonnet	Max
Are the aims of the publication clear?	4.4	4.2	5
Does it achieve its aims?	4.3	4.1	5
Is it relevant?	4.6	4.5	5
Is it clear what sources of information were used?	3.8	3.9	5
Is it clear when the information was produced?	2.9	3.0	5
Is it balanced and unbiased?	4.5	4.3	5
Does it provide details of additional sources?	3.6	3.7	5
Does it refer to areas of uncertainty?	4.2	4.4	5
Does it describe how each treatment works?	4.3	4.2	5
Does it describe the benefits of each treatment?	4.1	4.0	5
Does it describe the risks of each treatment?	4.4	4.1	5
Does it describe what would happen if no treatment is used?	4.0	4.0	5
Does it describe how the treatment choices affect overall quality of life?	4.4	4.2	5
Does it make clear that there may be more than one treatment choice possible?	4.3	4.2	5
Does it provide support for shared decision making?	4.7	4.8	5
Based on the answers to the 15 questions above, how would you rate the overall quality of each chatbot as a source of information about treatment choices?	4.4	4.3	5
TOTAL (Mean Composite)	4.2	4.1	5

Scores range from 1 (poor quality) to 5 (excellent quality). Composite scores represent mean ratings across all 25 responses per model averaged between nephrologists. Sub-domain items adapted from Charnock et al. (1999) (9).

### 3.6 Clinical appropriateness and safety communication

Both models consistently recommended a nephrologist or primary care consultation before initiating a fasting regimen: ChatGPT-5 included this recommendation in 92% of responses, and Claude 4.5 Sonnet in 88%. None of the responses generated by either chatbot were deemed clinically dangerous. Both chatbots appeared to avoid specifications regarding a fasting regimen or adjustments to medication without proper clinical evaluation individualized to the patient.

### 3.7 Response style

Qualitative differences in response style were apparent between models. ChatGPT-5 generated shorter, more structured responses organized around concise safety points, frequently using bullet formats. Claude 4.5 Sonnet produced longer, more discursive responses with detailed mechanistic explanations and broader contextualization of evidence limitations. The former style may be more accessible for patients with limited health literacy, and the latter may better serve patients seeking comprehensive background information.

## 4 Discussion

This study provides a structured evaluation of ChatGPT-5 and Claude 4.5 Sonnet on patient education questions about IF in CKD, a clinical topic where published guidance remains sparse. Both chatbots were accurate and consistent across most topics with improved response quality in domains with more published evidence such as electrolyte disturbances, hypoglycemia, and cardiovascular considerations. Our findings indicate AI chatbots may be utilized as a supplemental educational tool for patients with CKD if there is appropriate clinical oversight by a physician.

This study builds upon prior work by Acharya et al. evaluated AI chatbot accuracy for general CKD lifestyle and nutrition questions generated from KDIGO and KDOQI guidelines utilizing ChatGPT 3.5, ChatGPT-4, and Bard AI (12). The present study differs in several respects: it focuses specifically on IF, a dietary intervention not covered by existing CKD dietary guidelines, includes Claude 4.5 Sonnet as a comparator platform not previously evaluated in this context, applies the DISCERN instrument for health information quality assessment, and employs a repeated submission design to probe consistency. Taken together, these differences make the present study a meaningful extension of that earlier work. In addition to the work by Acharya et al, a cross-sectional study by Han et al. examined 216 adults in the US with CKD including kidney transplant recipients across several domains including preference, empathy, overall learning outcomes, and quality of information (16). In this study, generative AI platforms including ChatGPT were overall found to properly educate and engage users on kidney transplant -related topics provided by the National Kidney Foundation (NKF) though it should be noted that the reference literature cited is well established when compared to our study.

The readability difference between models is clinically relevant. The mean FKGL of 8.5 for ChatGPT-5 places its responses within the range most appropriate for the general US adult population, where health literacy averages approximately the 6^th^ to 8^th^ grade level (13). Claude 4.5 Sonnet’s mean FKGL of 10.2 may represent a barrier for a portion of the CKD population, particularly elderly patients, less educated individuals, and nonnative English-speaking groups that are overrepresented among patients with advanced CKD (17, 18). Neither model achieved the 6^th^ to 7^th^ grade reading level target often recommended for patient-facing health materials, indicating room for improvement in both platforms (19). The patterns of this study are consistent with previous studies examining health information generated by AI. Ensari et al. identified similar findings in their assessment of large language models in pediatric dialysis and additionally identified similar recurrent limitations across platforms (20). A parallel study by Ensari et al. evaluating AI based information consistency regarding pediatric urolithiasis also demonstrated similar findings consistent with the known accuracy and readability of current AI large language models also found in our study (21).

The improvement in accuracy scores between sessions, most evident for ChatGPT-5 (42/50 to 46/50) and to a lesser degree for Claude 4.5 Sonnet (42/50 to 44/50), requires some caution in interpretation. Since neither chatbot retains user session data by default under standard use conditions, this likely reflects the probabilistic nature of large language model output rather than any form of adaptive learning. This has been observed in other AI chatbot evaluation studies and may partly reflect the nature of response generation rather than memory or feedback (22, 23). Clinicians and patients should be aware that the same chatbot may produce meaningfully different responses to the same question across sessions.

One of the more practically useful findings was that both models reliably acknowledged the scarcity of CKD-specific evidence on IF. Recognizing the boundaries of available evidence, and communicating those limits clearly to patients, is an important feature of safe health communication. Both models appropriately cited the need for clinical evaluation by a medical professional rather than providing guidance in a medically complex population and topic with limited literature rather than extrapolating pre-existing evidence with little applicability. This chatbot behavior, while beneficial within the context of IF and CKD, should remain a concern for clinicians when discussing dietary guidance with patients.

The ‘obesity paradox’ in dialysis patients warrants acknowledgement in our study. Among the general population obesity is a well-known contributor to worsening cardiovascular morbidity and mortality with weight loss encouraged to decrease overall disease risk (24). Paradoxically however, multiple studies have shown improvements in overall survival metrics among CKD patients with higher BMI undergoing hemodialysis (25, 26). In these studies, a low BMI (< 18.5 kg/m^2^) was associated with increased mortality. The ‘obesity paradox’ stands in stark contrast to pre-existing notions of obesity and increases or decreases in cardiovascular risk factors, though it should be noted that this only applies to dialysis dependent CKD patients. None of the AI generated responses mentioned the obesity paradox, likely because it is missing from current guidelines, nonetheless, it is an important concept that must be addressed in both future studies and must be incorporated by AI when educating CKD patients, particularly those on dialysis, on IF (27). Neither chatbot was specifically tested on this topic and it is entirely plausible that AI platforms are trained on both general population dietary literature and specific subgroup evidence, thus future evaluations should address this important gap.

Many of the weaker subject response performances by both chatbots, as evidenced in **Table 3**, mirrors real world deficiencies in the literature rather than AI chatbot shortcomings. Subjects including sex-based differences in IF response in CKD, long-term outcomes of IF in CKD, and fasting during dialysis have limited published systematic study. As dietary research in patients with CKD expands, AI chatbot performance is expected to improve but clinicians must remain aware of the potential for inaccuracies by AI platforms and inform their patients on the dangers of utilizing AI platforms for primary medical guidance rather than as a supplemental tool.

The consistently low DISCERN sub-domain scores for ‘information source citation’ and ‘timeliness’ reflect a structural limitation of current AI chatbot platforms: neither model cites primary literature or discloses its training data. Independent claims and comparisons to current guidelines can therefore not be independently verified by patients. AI chatbots should incorporate logged and real-time literature retrieval with citations to address such a limitation. Response reliability, even with resolution of such a limitation, should still be discussed with a licensed nephrologist or other clinician.

The study had several limitations. First, response accuracy was assessed by two evaluators which, while having similar overall scores, did not undergo standardized inter-rater assessment which is a methodological limitation. It should be noted however that steps were made to limit subjectivity in the absence of a standardized multi-rater protocol, as literature was utilized only if PubMed indexed with a significant number of questions paired to current guidelines from KDIGO or KDOQI, the leading worldwide authorities in the field of CKD. Future studies should employ a structured consensus approach with formal inter-rater reliability metrics. Additionally, future studies would benefit from a blinded multi-rater design with the Cohen’s kappa coefficient statistic to assess for inter-rater reliability. Second, the 25-question set, though systematically developed, covers a curated sample of clinical topics and may not represent the full range of queries patients generate in practice. Third, responses were evaluated from static, single-turn submissions; real-world patient interactions with AI platforms frequently involve multi-turn conversations that may yield different outcomes. Fourth, AI platforms undergo continuous version updates, and the performance characteristics reported here reflect ChatGPT-5 and Claude 4.5 Sonnet at the time of this study. Reassessment as platforms are updated will be needed. Fifth, this study measured information quality and readability but did not assess patient comprehension, behavioral response, or downstream clinical outcomes following AI-assisted education, outcomes that would be necessary to establish clinical utility. Sixth, though the goal was to mimic patient-centered language in each question, the questions themselves were generated by clinicians. Despite attempts to relay lay language, the clinician generated prompts may have differences from the natural phrasing of patients regarding health inquiries and thus overestimate clarity and clinical precision of AI interactions with patients. Future studies examining AI health-based interactions with patients should seek to incorporate questions specifically from the applicable population to reflect authenticity.

Though our study had two raters, no formal inter-rater evaluation was performed, rather, the composite scores of the nephrologists were averaged and reported. Future research should include multi-evaluator rubric designs with standardized inter-rater reliability reporting, evaluation of multilingual response quality for non-English-speaking CKD populations, and longitudinal assessment of chatbot performance across model updates. Patient-centered outcome studies examining whether AI-assisted dietary education translates into improved understanding, dietary adherence, or clinical outcomes in CKD would substantially strengthen the evidence base.

## 5 Conclusion

ChatGPT-5 and Claude 4.5 Sonnet both produce clinically accurate, consistent, and appropriately cautious responses to patient questions about IF in CKD, supporting a potential supplementary educational role for these platforms. ChatGPT-5 responses are better aligned with US adult health literacy norms. Both chatbots conveyed the appropriate recognition of limited literature regarding IF and CKD and consistently recommended physician counseling or oversight. Specific subject related weaknesses, most pronounced for sex-based IF differences, long-term CKD outcomes, and dialysis-specific guidance, reflect real gaps in published literature rather than AI-specific problems. AI chatbots should serve as informational supplements to and not replacements for individualized nephrology counseling, particularly for complex dietary decisions in medically vulnerable patient populations with limited evidence.

## Data Availability

The original contributions presented in the study are included in the article/**Supplementary Material**. Further inquiries can be directed to the corresponding author.
