# Supplementary material for: Accuracy, readability, and health information quality of AI chatbot responses for patient education on intermittent fasting in chronic kidney disease: a prospective structured evaluation
Source: Front Nephrol. 2026 May 29;6:1857793. doi: 10.3389/fneph.2026.1857793 (PMC13259986; doi:10.3389/fneph.2026.1857793)
Supplement: Supplementary file 1 [file SupplementaryFile1.docx]

Supplementary Appendix: Complete Question Set

The following 25 patient-centered questions were developed and submitted to ChatGPT-5 and Claude 4.5 Sonnet:

1. Is intermittent fasting safe for someone like me with chronic kidney disease?

2. Can intermittent fasting make my kidney function worse?

3. Are some types of fasting safer or better for people with kidney disease than others?

4. How does fasting affect my body's fluid levels and electrolytes?

5. Can fasting change how much protein is leaking into my urine?

6. Could fasting make my kidney disease get worse faster or slow it down?

7. Does fasting help or hurt my blood pressure?

8. Is fasting safe if I have both kidney disease and diabetes?

9. Is fasting safer in early kidney disease compared to later stages?

10. If I'm on dialysis, can I still fast, and how should I do it safely?

11. What should I pay attention to in my diet if I'm fasting with kidney disease?

12. Can fasting increase my risk of high potassium levels?

13. Does fasting change how my medications work in my body?

14. How might fasting affect my mood, stress, or daily life?

15. Can fasting make problems like anemia better or worse?

16. Do we know what happens long-term if people with kidney disease fast?

17. How should my doctor and I monitor things if I decide to try fasting?

18. Can fasting affect acid levels in my body?

19. Are there situations where fasting would not be safe for me at all?

20. How does fasting affect my heart health if I have kidney disease?

21. Do men and women respond differently to fasting with kidney disease?

22. Does the timing of fasting (like how long or what time of day) matter for safety?

23. What's the best way for me to learn how to fast safely with kidney disease?

24. Does fasting affect my gut health, and does that matter for my kidneys?

25. Is it always appropriate for doctors to recommend fasting to patients like me?
